# A dual-drug chitosan–hyaluronic acid bandage contact lens accelerates corneal re-epithelialization in a rabbit mechanical debridement model

**DOI:** 10.1038/s41598-026-51386-1

**Published:** 2026-05-07

**Authors:** Mohamadreza Aghamirsalim, Erfan Sadeghi, Mahmood Jabbarvand, Farshad Famildardashti, Farzad Famildardashti, Alireza Sahraian

**Affiliations:** 1 Translational Ophthalmology Research Center, Tehran University of Medical Sciences, Tehran, 1417614411 Iran; 2 Department of Biostatistics, School of Medicine, Shiraz University of Medical Sciences, Shiraz, Iran

**Keywords:** Bandage contact lens, Betamethasone, Chitosan, Corneal epithelial healing, Drug-loaded hydrogel, Hyaluronic acid, Levofloxacin, Rabbit model, Diseases, Drug discovery, Medical research

## Abstract

Rapid corneal re-epithelialization is crucial to prevent infection and scarring, yet the efficacy of conventional topical therapies is hindered by their limited ocular bioavailability. To address this, we developed a daily replaceable chitosan–hyaluronic acid (CS–HA) bandage contact lens co-loaded with levofloxacin and betamethasone using a soaking-based drug-loading approach. In a rabbit 8-mm mechanical corneal debridement model (*n* = 15/group), the dual-drug CS–HA lens was compared with a chitosan-only drug-loaded lens, a commercial silicone-hydrogel bandage lens plus drops, and drops alone. The CS–HA lens maintained adequate optical transparency, water content, and oxygen permeability suitable for short-term wear. In vitro assays showed that the dual-drug CS–HA lens retained antibacterial inhibitory activity in agar diffusion tests and reduced TNF-α secretion in an LPS-stimulated THP-1 assay. Re-epithelialization was significantly faster with the CS–HA lens, achieving complete closure at a median of 3 days versus 5, 6, and 8 days in the respective control groups. Day-10 histology showed a well-stratified epithelium with no evidence of stromal inflammatory infiltration in the CS–HA group, whereas controls exhibited greater stromal disorganization. This biocompatible dual-drug platform may accelerate ocular surface restoration and reduce reliance on frequent topical dosing in settings such as post-refractive surgery care and persistent epithelial defects.

## Introduction

The corneal epithelium plays a critical role in maintaining ocular surface integrity and visual clarity. Rapid and orderly re-epithelialization after injury is essential to restore barrier function, prevent infectious keratitis, and minimize stromal complications that can compromise vision^1^. Epithelial defects occur in diverse clinical contexts, including corneal cross-linking (CXL), photorefractive keratectomy (PRK), traumatic abrasions, chemical injuries, and ocular surface reconstruction, where delayed healing prolongs pain and increases the risk of infection, haze, and scarring^2–4^. In these conditions, the current standard of care consists of applying a bandage contact lens (BCL) to protect the corneal surface, combined with frequent instillation of topical antibiotics and corticosteroids^5–7^. However, conventional regimens suffer from poor patient compliance and critically low ocular bioavailability (less than 5%), necessitating innovative biomaterial-based drug-delivery systems for enhanced therapeutic efficacy^8^.

Drug-loaded contact lenses have recently emerged as an alternative to conventional drops, aiming to improve compliance and maintain therapeutic drug levels on the ocular surface^9^. Among emerging biomaterials, chitosan-hyaluronic acid combinations offer unique advantages for post-surgical applications. Chitosan provides optical transparency, mucoadhesion, and antimicrobial activity^10^, while hyaluronic acid accelerates epithelial migration and stabilizes the tear film^11^. Their synergistic interaction enhances drug retention, improves corneal penetration, and promotes healing through complementary mechanisms^12–14^.

In this study, we evaluated a bioengineered chitosan–hyaluronic acid bandage contact lens loaded with levofloxacin and betamethasone in a rabbit model of an 8-mm central mechanical epithelial debridement. We hypothesized that this platform would accelerate epithelial closure compared with standard care, reduce reliance on frequent topical medications, and minimize early complications.

## Methods

### Study design

This was a randomized, controlled, parallel-group preclinical study in New Zealand White rabbits comparing a daily replaceable CS–HA dual-drug bandage contact lens with three controls: a daily replaceable CS-only dual-drug lens, a daily replaceable commercial silicone-hydrogel BCL plus topical drops, and drops only. The protocol was approved by the Farabi Eye Hospital Ethics Committee (IR.TUMS.FARABIH.REC.1399.001; 27 May 2020) and adhered to the ARVO Statement for the Use of Animals in Ophthalmic and Vision Research; reporting follows ARRIVE 2.0^15,16^.

### Animals and housing

Sixty adult male New Zealand White rabbits (2.5–3.0 kg) were acclimatized for 7 days under controlled conditions (21 ± 2 °C; 50 ± 10% relative humidity; 12-h light/dark cycle). One eye per rabbit (randomly selected) was included; the fellow eye remained untreated. Animals with any ocular abnormality at screening were excluded.

### Randomization and group allocation

Block randomization (block size = 4) allocated 60 eyes equally into four groups (A–D; *n* = 15 per group). Group assignments were concealed in sequentially numbered, opaque, sealed envelopes and revealed only after baseline imaging. Laterality of the study eye (right or left) was determined by coin flip. Outcome assessment was image-based: fluorescein-stained corneal photographs were exported with anonymized filenames, and two masked graders quantified epithelial defect area; discrepancies were adjudicated by a third masked observer. Data analysts remained masked until database lock. The treatment groups were defined as follows: (i) Group A (CS–HA lens): Chitosan–hyaluronic acid composite lens co-loaded by soaking in levofloxacin 0.5% (w/v) and betamethasone sodium phosphate 0.1% (w/v) solutions (hereafter referred to as CS-HA-Beta-Levo). (ii) Group B (CS lens): Chitosan-only lens co-loaded using the same soaking solutions (levofloxacin 0.5% w/v and betamethasone sodium phosphate 0.1% w/v). (iii) Group C (Commercial BCL): Silicone-hydrogel bandage lens (Air Optix Night & Day Aqua, Alcon, Fort Worth, TX, USA) plus Topical levofloxacin 0.5% (Santen Pharmaceutical Co., Osaka, Japan) and betamethasone 0.1% (Shionogi & Co., Osaka, Japan) were administered q8h. (iv) Group D (Drops only): Topical levofloxacin 0.5% and betamethasone 0.1% (q8h) without a lens.

### Contact lens preparation

Chitosan (≥ 75% deacetylation; Sigma-Aldrich, Steinheim, Germany) and high-molecular-weight hyaluronic acid (HMW-HA, 1.5–1.8 MDa; Sigma-Aldrich, St. Louis, MO, USA) were prepared as 2% (w/v) chitosan in 1% (v/v) acetic acid and 1% (w/v) HA in sterile deionized water, each stirred overnight at room temperature (RT). Equal volumes were blended (1:1, v/v) to yield a 1.5% (w/v) casting solution (final concentrations: chitosan 1.0% and HA 0.5% w/v), degassed, and cast into contact lens–shaped PTFE molds (14-mm diameter; concave posterior surface) in three 900-µL layers with partial drying between layers (30–45 min, laminar flow). Casts were air-dried for 24 h at RT, neutralized in 0.1–0.25 M NaOH for up to 10 min to achieve pH 7.0–7.4, and rinsed three times with sterile phosphate-buffered saline (PBS). After hydration in PBS, the constructs formed flexible, lens-shaped hydrogels that conformed to the corneal surface for short-term bandage wear. Acetic acid, NaOH, and PBS were purchased from Merck KGaA (Darmstadt, Germany). Low-viscosity aqueous solutions (e.g., PBS and drug solutions) were sterilized by 0.22-µm filtration. Chitosan and hyaluronic acid precursor solutions were prepared under aseptic conditions. Dried constructs underwent ethylene-oxide sterilization followed by 48–72 h aeration in accordance with ISO 10993-7^17,18^. For the chitosan-only lens (Group B), the same casting, drying, neutralization, hydration, and sterilization workflow was used, except that hyaluronic acid was omitted. Drug loading was performed after neutralization and hydration by immersing the lenses in a sterile, preservative-free mixed aqueous solution prepared from analytical-grade levofloxacin powder (0.5% w/v) and betamethasone sodium phosphate powder (0.1% w/v), using a conventional soaking-based loading method for soft hydrogel lenses^19,20^. Each lens specimen was submerged in 3.0 mL of the drug solution for 24 h at room temperature (25 °C) under orbital agitation (50 rpm). After incubation, the lenses were briefly rinsed with sterile PBS to remove loosely bound surface residues and were implanted immediately in the hydrated state. All post-sterilization handling and drug-loading procedures were performed aseptically in a laminar-flow hood using sterile containers and solutions. The dry thickness (~ 0.20 mm) was confirmed with a calibrated micrometer (*n* ≥ 10). Absolute drug content per lens was not quantified in the present study.

### Lens physicochemical characterizations

Optical transmittance (200–1000 nm, 1-nm steps) was measured on a double-beam UV-visible spectrophotometer (Shimadzu, Kyoto, Japan) with lenses immersed in saline at 25 °C and after blot-drying in air, following ISO 18369-4 guidance^21^. Equilibrium water content (EWC) was determined gravimetrically after 7-day saline equilibration; refractive index was estimated from EWC according to González-Méijome et al.^22^. Oxygen permeability (Dk) was measured at 35 ± 1 °C using a polarographic stack method (Model 201T; Createch, Albany, CA, USA), with lens stacks spanning ~ 200–600 μm; the inverse apparent transmissibility was regressed on thickness to derive Dk^23^.

### In vitro functional bioactivity assays

To verify the functional activity of the released levofloxacin, a direct agar diffusion bioassay was performed. *Staphylococcus aureus* (Gram-positive) and *Escherichia coli* (Gram-negative) were cultured on Mueller-Hinton agar plates using a 0.5 McFarland standard inoculum. The drug-loaded hydrogel specimens (CS-HA-Beta-Levo) were cut into uniform pieces and placed directly onto the inoculated agar surface. Drug-free bandage contact lenses of identical dimensions were placed on the same plates to serve as internal negative controls. After 24 h of incubation at 37 °C, the functionality of the released antibiotic was evaluated by measuring the diameter of the clear zones of inhibition around the samples using a digital caliper. All tests were performed in three independent biological replicates (*n* = 3).

To assess the anti-inflammatory bioactivity of the dual-drug lens eluate, conditioned eluates were generated by incubating one CS-HA-Beta-Levo lens in 2.0 mL of sterile PBS at 37 °C for 24 h. Human THP-1 monocytes were differentiated into macrophage-like cells using 50 ng/mL phorbol 12-myristate 13-acetate (PMA) for 48 h prior to the assay. The differentiated cells were stimulated with 100 ng/mL lipopolysaccharide (LPS) and concurrently treated with undiluted lens eluates. After incubation, anti-inflammatory activity was quantified by measuring tumor necrosis factor-alpha (TNF-α) secretion using a commercial ELISA kit. For comparison across independent biological replicates (*n* = 3), the TNF-α concentration in the LPS-only group was normalized to 100%, and all other values were expressed as relative percentages.

### Surgical procedure

Anesthesia was induced with intramuscular ketamine (35 mg/kg) and xylazine (5 mg/kg) (Alfasan, Woerden, The Netherlands). After topical anesthesia with 0.5% proparacaine hydrochloride (Alcon Laboratories, Fort Worth, TX, USA), an 8-mm central corneal epithelial debridement was created using a trephine and sterile spatula. No stromal ablation was performed. Immediately thereafter, group-specific treatments were applied as described above. Postoperative analgesia consisted of subcutaneous buprenorphine (0.05 mg/kg) (Reckitt Benckiser, Slough, UK) every 12 h for 48 h.

### Daily lens replacement and imaging protocol

To permit accurate daily assessment of the corneal epithelial defect (CED) area without optical interference or nonspecific fluorescein retention by the hydrogel matrix, lenses in Groups A, B, and C were briefly removed before fluorescein instillation at each scheduled imaging session. Lens removal was performed under topical anesthesia by trained personnel using sterile blunt forceps and aseptic technique. After slit-lamp examination and standardized fluorescein imaging, the removed lens was discarded and a new sterile lens from the corresponding treatment group was immediately applied. For Groups A and B, replacement lenses were freshly drug-loaded using the same 24-h soaking protocol before application. For Group C, a new sterile commercial silicone-hydrogel bandage lens was applied after each imaging session. No removed lens was reused. Lens position, retention, and ocular surface tolerance were evaluated daily.

### Outcomes and assessments

**Fig. 1:**
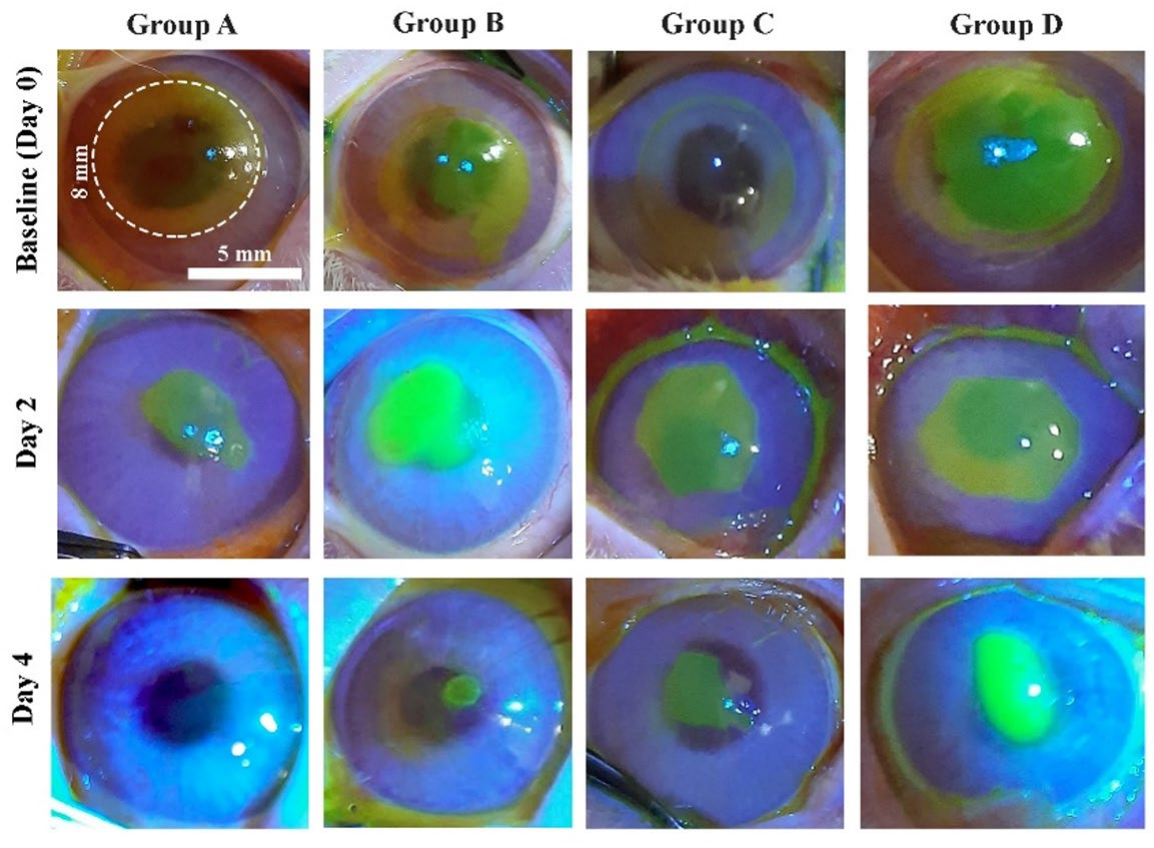
Serial fluorescein photographs after standardized 8‑mm epithelial debridement. Columns correspond to Groups **A**–**D**, and rows indicate Baseline (Day-0), Day-2, and Day-4. The dashed circle indicates the initial 8-mm defect size (Scale bar = 5 mm). Green fluorescein delineates the residual epithelial defect, illustrating progressive reduction in each group.

Corneal epithelial defect (CED) area was assessed at baseline (immediately after debridement), at 24, 48, and 72 h, and then daily through day-10. After topical anesthesia, 1% sodium fluorescein (Sigma-Aldrich, St. Louis, MO, USA) was instilled and slit-lamp photographs were obtained using a slit-lamp biomicroscope (Haag-Streit, Koeniz, Switzerland) under cobalt-blue illumination with a yellow barrier filter using standardized imaging parameters. In lens-treated groups, imaging was performed after brief removal of the existing lens and before application of the new replacement lens. CED area (mm²) was quantified in ImageJ software (version 1.54s; National Institutes of Health, Bethesda, MD, USA; https://imagej.net/ij/) according to the method of Hashemi et al.^24^. Calibration was performed using an in-frame scale reference in the same focal plane. To ensure measurement accuracy, CED area was quantified using a dual-masked grading protocol. Two independent masked observers contoured the defects using the same calibrated software workflow. In cases of inter-observer discrepancy (defined as > 5% variance), images were reviewed by a third senior masked adjudicator to establish the final consensus value used for analysis. Complete re-epithelialization was defined as the first absence of fluorescein staining (Fig. 1). The primary (confirmatory) outcome measure was the time to complete re-epithelialization. Analyses of CED area at individual time points were prespecified as secondary (exploratory) outcomes to describe the healing trajectory.

At 10 days post-treatment, animals were humanely euthanized by intravenous administration of an overdose of sodium pentobarbital (> 100 mg/kg), followed by tissue collection from six randomly selected eyes per group (Groups A–D; 6 of 15 per group). Death was confirmed by the absence of spontaneous respiration and heartbeat (thoracic auscultation) and loss of brainstem reflexes, including the corneal blink response. Samples were fixed in 10% neutral-buffered formalin, embedded in paraffin, and sectioned at 5 μm. Hematoxylin and eosin (H&E) staining was performed, and sections were evaluated for epithelial organization, stromal inflammatory cell infiltration, and evidence of neovascularization or other pathological alterations using a light microscope (Olympus, Tokyo, Japan) (Fig. 5).

Daily clinical examinations were performed to monitor for infection, inflammation, corneal opacity, neovascularization, or discomfort.

### Sample-size calculation

Power analysis was performed for three prespecified comparisons with the drops-only control (A vs. D, B vs. D, C vs. D). Assuming a clinically meaningful 2-day reduction in re-epithelialization time with SD ≈ 1.2 days, we set familywise α = 0.05 using Bonferroni correction (per-comparison α = 0.0167) and targeted 80% power. The calculation indicated a minimum of 10 eyes per group (one eye per rabbit). To enhance robustness and cover potential attrition, we enrolled 15 eyes per group, thereby exceeding the required sample size.

### Statistical analysis

Continuous variables are summarized as mean ± standard deviation (SD). For the in vitro functional assays, group differences for each outcome were analyzed separately using one-way ANOVA followed by Tukey’s post hoc test. Time-to-heal is additionally summarized as median (IQR) and analyzed as a time-to-event outcome. Baseline CED area (day-0) was compared across groups using one-way ANOVA. For the day-3 CED comparison, a baseline-adjusted linear model was fitted using an ANCOVA approach implemented with the GENLIN procedure in IBM SPSS Statistics (version 25.0; IBM Corp., Armonk, NY, USA; https://www.ibm.com/support/pages/ibm-spss-statistics-25-documentation), with a normal distribution and identity link, treatment group as a fixed factor, and baseline CED (day-0) as a covariate. Bonferroni correction was applied for the three prespecified comparisons versus the drops-only control (Group D) (α = 0.05/3 = 0.0167). Both unadjusted and Bonferroni-adjusted p-values are reported where applicable. Day-3 was used for between-group CED comparisons because a floor effect was observed by day-4. Time to complete re-epithelialization (primary endpoint) was analyzed using Kaplan–Meier methods and compared among groups with the log-rank (Mantel–Cox) test. Eyes requiring protocol-deviating rescue therapy (e.g., intensified antimicrobials for infectious keratitis) were excluded from the primary time-to-event (Kaplan–Meier/log-rank) analysis; CED measurements obtained prior to rescue therapy were retained for longitudinal/secondary analyses. Pairwise log-rank comparisons were performed for the six group-to-group contrasts, with Bonferroni correction applied for multiplicity (α = 0.05/6 = 0.0083). All tests were two-sided, and exact p-values are reported. Statistical significance was set at *p* < 0.05 unless otherwise specified after multiplicity correction.

### Ethical considerations and humane care

All procedures adhered to approved protocols; animals were monitored at least daily for infection, inflammation, corneal opacity, neovascularization, and distress. Humane endpoints and veterinary oversight were in place. Analgesia was provided as above.

## Results

### Physicochemical properties of the CS–HA Lens

**Fig. 2:**
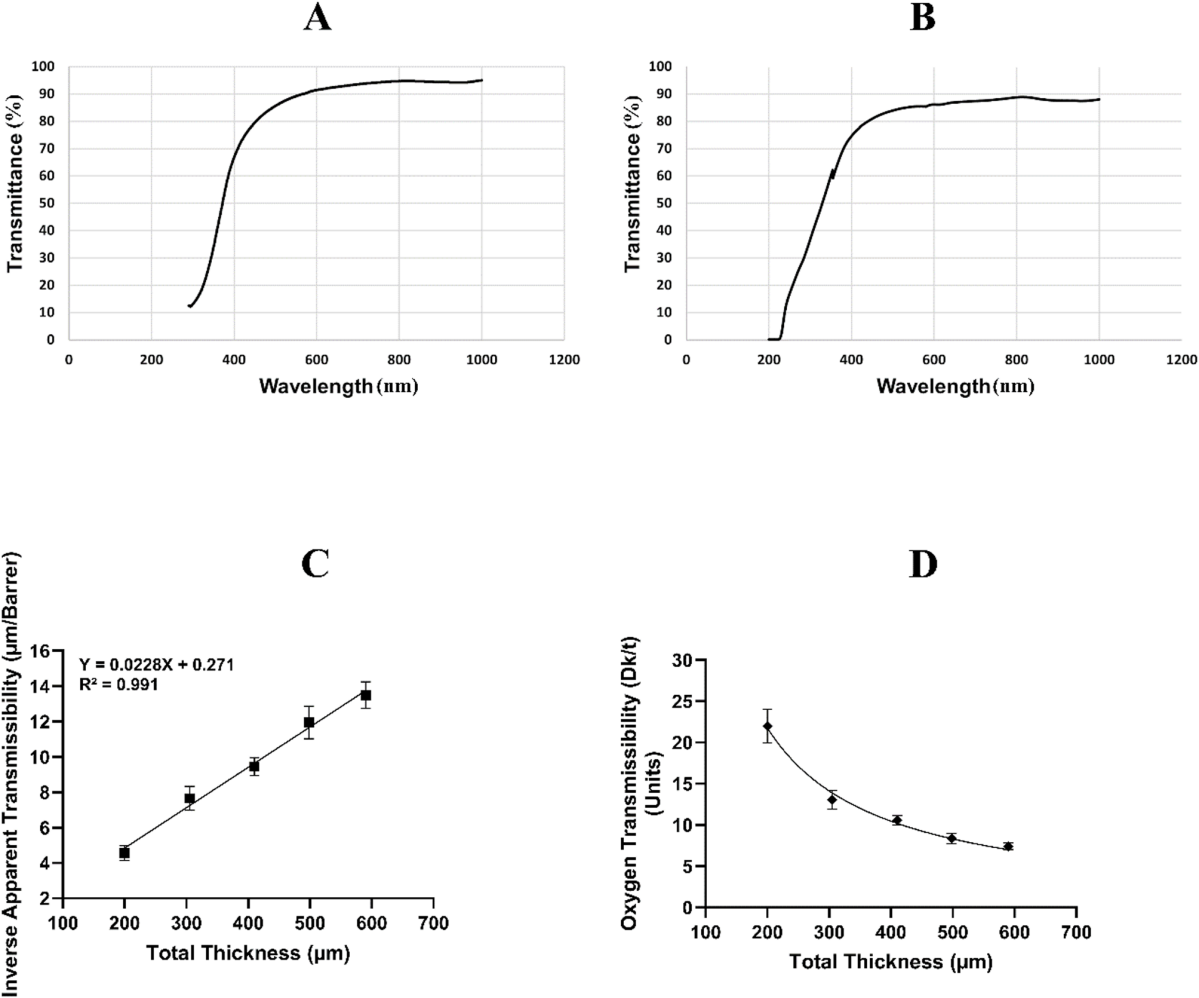
Physicochemical characterization of the CS–HA lens. (**A**) UV–visible transmittance of the hydrated lens in saline. (**B**) UV–visible transmittance of the blot-dried lens. (**C**) Determination of intrinsic oxygen permeability (Dk). Data points represent mean ± SD (*n* = 5); the solid line indicates the linear regression fit (Y = 0.0228X + 0.271; R² = 0.991). (**D**) Oxygen transmissibility (Dk/t) profile versus thickness, with the curve representing the theoretical hyperbolic fit derived from the linear regression parameters.

Spectrophotometry showed limited UV-B transmission at 295 nm (12.3%) and partial UVA/short-wavelength visible transmission at 385 nm (59.4%) and 400 nm (66.8%). Accordingly, visible-light transmittance reached ≥ 90% specifically from 567 nm onwards and plateaued at ~ 94.5% up to 780 nm, indicating that optimal transparency is restricted to the mid-to-long wavelength region of the visible spectrum (Fig. 2A, B). EWC was 79.8 ± 2.1%, corresponding to a mass swelling ratio of approximately 3.95 (calculated as (W_wet - W_dry) / W_dry), with an estimated refractive index of 1.37 ± 0.01. Oxygen permeability (Dk) was determined using the polarographic stack method. Linear regression analysis revealed a highly significant correlation (R² = 0.991) between inverse apparent transmissibility and total specimen thickness (Fig. 2C). The regression slope was 0.0228 ± 0.0012 (SE) Barrer⁻¹, corresponding to an intrinsic oxygen permeability (Dk) of 43.9 ± 2.4 Barrer (1.47 × 10⁻¹⁴ mol m⁻¹ s⁻¹ Pa⁻¹). The y-intercept was determined to be 0.271 ± 0.528 (SE), representing the estimated boundary layer resistance. Based on these regression parameters, the oxygen transmissibility (Dk/t) profile was plotted, exhibiting the expected hyperbolic decrease with increasing lens thickness (Fig. 2D).

### Functional bioactivity of the CS–HA Lens

**Fig. 3:**
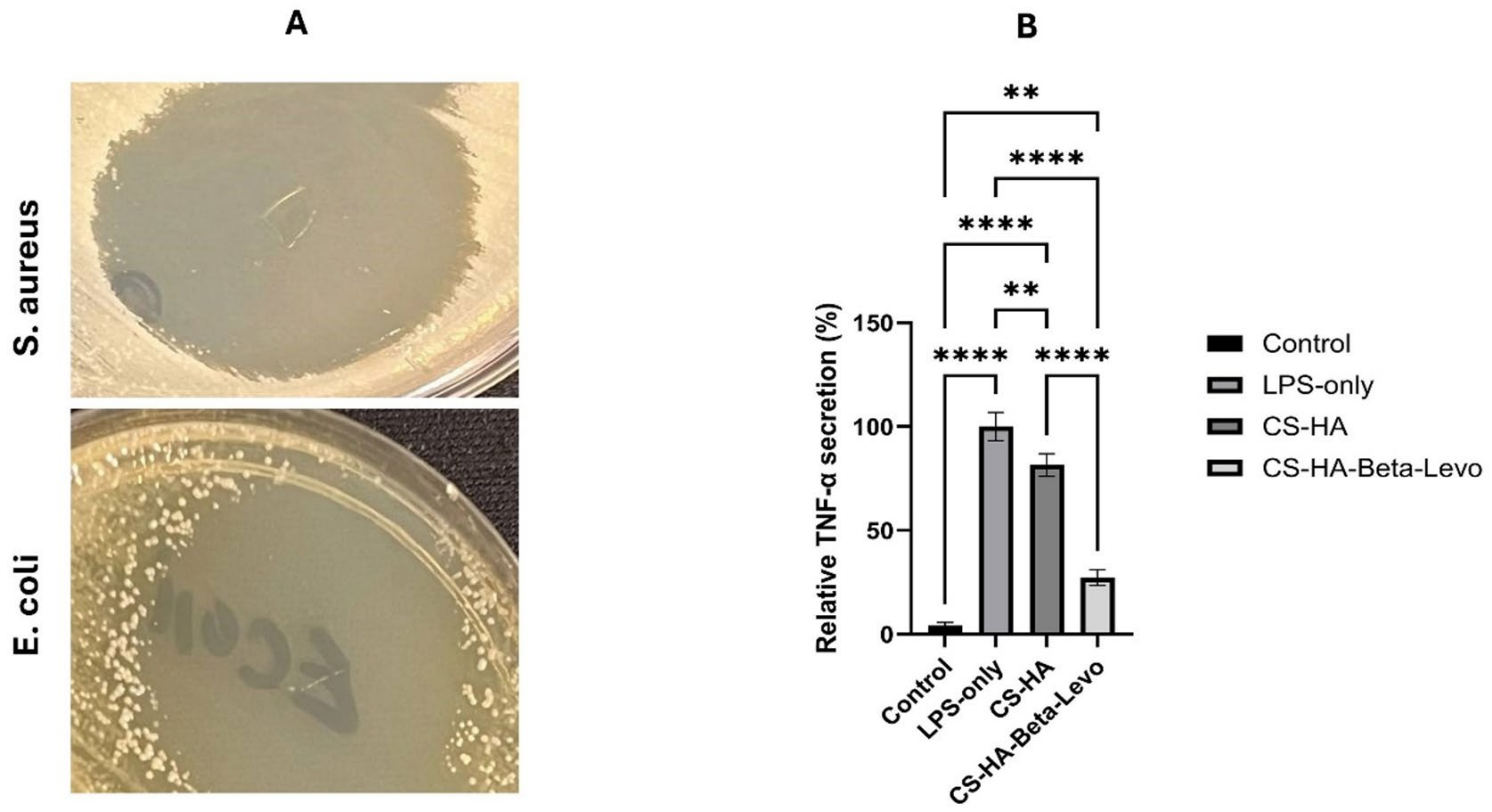
In vitro functional bioactivity of the CS–HA lens. (**a**) Antibacterial activity against *S. aureus* and *E. coli* showing zones of inhibition. (**b**) Anti-inflammatory bioactivity measured via relative TNF-α secretion (%) from LPS-stimulated macrophages. Relative TNF-α secretion levels are shown following treatment with the drug-free CS-HA matrix and the CS-HA-Beta-Levo lens eluate. Data are presented as mean ± SD (*n* = 3). **** *p* < 0.0001 and ** *p* < 0.01 based on one-way ANOVA with Tukey’s *post hoc* test.

The dual-drug CS-HA lens produced inhibition zones of 33 ± 1 mm against *Staphylococcus aureus* (*S. aureus*) and 31 ± 1 mm against *Escherichia coli* (*E. coli*) (Fig. 3a). In LPS-stimulated macrophages (normalized to 100% TNF-α secretion), treatment with the drug-free CS-HA matrix eluate resulted in a relative TNF-α secretion of 81.5 ± 5.4%. Treatment with the dual-drug loaded eluate reduced TNF-α secretion to 27.3 ± 3.8% (*p* < 0.0001 compared to both LPS-only and CS-HA groups) (Fig. 3b).

### Corneal epithelial healing (clinical images and CED Area)

**Fig. 4:**
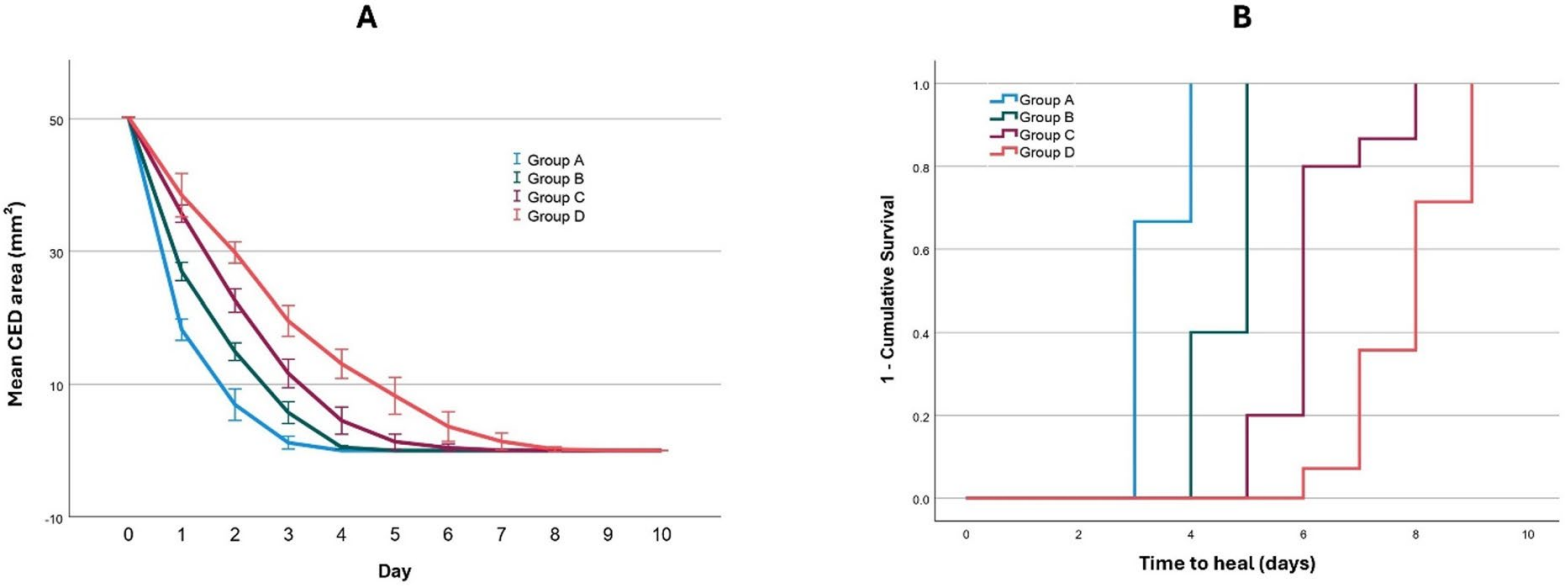
Quantitative assessments of corneal wound closure. (**A**) Line plot showing the trend of CED during follow-up, with error bars representing 95% confidence intervals. (B) Kaplan–Meier plot (1 minus cumulative survival) representing time to heal across treatments.

Serial fluorescein images documented progressive reduction in CED across all groups (Fig. 1). Baseline (day-0) CED did not differ among groups (one-way ANOVA *p* = 0.713). Because values in Group A (CS–HA) reached the floor by day-4, between-group comparisons focused on day-3 and were baseline-adjusted using an ANCOVA-type linear model. Compared with drops alone (Group D), baseline-adjusted day-3 CED areas were smaller in Group A (*p* = 4.38 × 10⁻⁵⁵; Bonferroni-adjusted *p* = 1.31 × 10⁻⁵⁴), Group B (*p* = 5.20 × 10⁻³²; Bonferroni-adjusted *p* = 1.56 × 10⁻³¹), and Group C (*p* = 2.97 × 10⁻¹¹; Bonferroni-adjusted *p* = 8.91 × 10⁻¹¹) (Table 1). Detailed coefficients and mean changes are presented in Table 1, and the healing trajectory is illustrated in Fig. 4A.

**Table 1:** Baseline-adjusted comparison of CED area at day-3 (ANCOVA-type). Baseline CED (day 0) was included as a covariate. β coefficients represent the adjusted difference in day-3 CED area (mm²) compared with Group D (drops only; reference). Unadjusted p-values are from Wald χ² tests (df = 1). Bonferroni-adjusted p-values account for three prespecified comparisons versus Group D (p_adj = min[*p* × 3, 1]).

Treatment	β (95% CI)	Unadjusted *p* (Wald χ²)	Adjusted *p*-value (Bonferroni)	Change ^*^
Group A (CS–HA lens)	-18.36 (-20.66, -16.06)	4.38 × 10⁻⁵⁵	1.31 × 10⁻⁵⁴	-49.09 ± 1.75
Group B (CS lens)	-13.84 (-16.15, -11.54)	5.20 × 10⁻³²	1.56 × 10⁻³¹	-44.55 ± 2.97
Group C (Commercial BCL + drops)	-7.85 (-10.17, -5.54)	2.97 × 10⁻¹¹	8.91 × 10⁻¹¹	-38.63 ± 3.87
Group D (Drops only)	Ref		Ref	-30.72 ± 4.20

*Change is mean ± SD change in CED area (mm²) from day 0 to day 3 (negative values indicate reduction).

### Time to complete re-epithelialization

Kaplan–Meier curves showed overall differences in healing time across groups (overall log-rank χ² = 82.066, df = 3, *p* = 1.11 × 10⁻¹⁷; Fig. 4B). Pairwise log-rank tests demonstrated significant differences across all six group-to-group contrasts (all unadjusted *p* ≤ 2.45 × 10⁻⁴; Table 2). After Bonferroni correction for six pairwise comparisons, all contrasts remained significant (maximum Bonferroni-adjusted *p* = 0.001471; Table 2). Median (IQR) time to heal was 3 (3–4) days for Group A, 5 (4–5) days for Group B, 6 (6–6) days for Group C, and 8 (7–9) days for Group D (Table 3). One eye in Group D developed bacterial keratitis requiring protocol-deviating intensified antimicrobial therapy and was excluded from the time-to-event (Kaplan–Meier/log-rank) analysis (Group D: *n* = 14).

**Table 2:** Pairwise log-rank comparisons for time to complete re-epithelialization.

Comparison	Log-rank χ²	Unadjusted *p*-value	Bonferroni-adjusted *p*†
Group A vs. Group B	19.460	1.0275 × 10⁻⁵	6.1651 × 10⁻⁵
Group A vs. Group C	31.069	2.4901 × 10⁻⁸	1.4941 × 10⁻⁷
Group A vs. Group D	29.590	5.3381 × 10⁻⁸	3.2029 × 10⁻⁷
Group B vs. Group C	20.766	5.1899 × 10⁻⁶	3.1140 × 10⁻⁵
Group B vs. Group D	27.030	2.0035 × 10⁻⁷	1.2021 × 10⁻⁶
Group C vs. Group D	13.449	2.4517 × 10⁻⁴	0.0014710

†Bonferroni adjustment was applied across six pairwise comparisons (p_adj = min[*p*×6, 1]). All comparisons remained significant under the Bonferroni-adjusted alpha of 0.0083 (0.05/6).

*Group D time-to-heal analysis included 14 eyes due to one protocol-deviating rescue treatment.

**Table 3:** Summary of time to complete re-epithelialization (days) in each group. Values are presented as mean ± SD and median (IQR). Time-to-heal was analyzed using Kaplan–Meier/log-rank methods.

Treatment	Mean ± SD	Median (IQR)
Group A	3.33 ± 0.49	3 (3–4)
Group B	4.60 ± 0.51	5 (4–5)
Group C	6.13 ± 0.92	6 (6–6)
Group D*	7.86 ± 0.95	8 (7–9)

* One eye in Group D was excluded from the time-to-event (Kaplan–Meier/log-rank) analysis due to protocol-deviating intensified treatment after bacterial keratitis (Group D *n* = 14).

### Histological findings

**Fig. 5:**
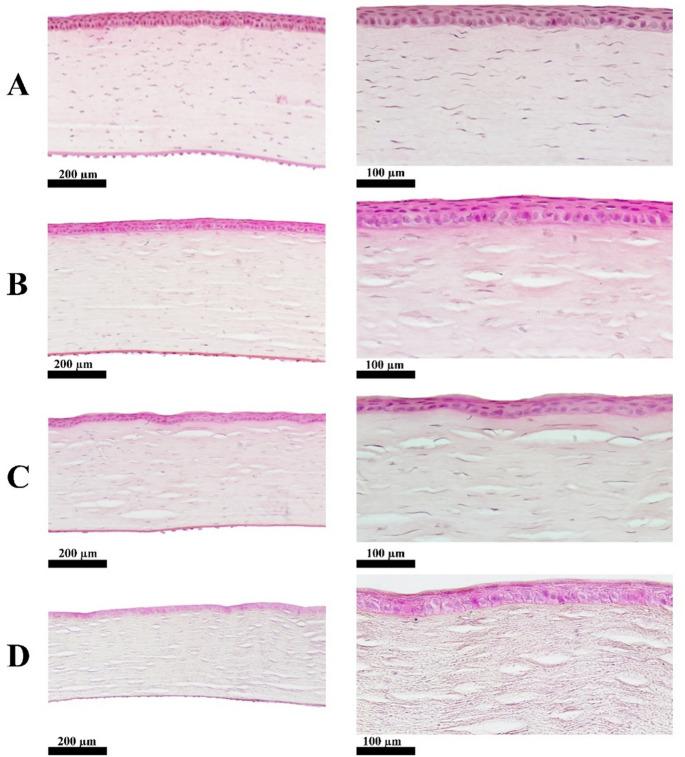
Representative histopathological sections (H&E staining) of corneal tissue at day-10 post-treatment, sampled from the central wound bed area. Images are shown at ×40 (left panels, scale bar = 200 μm) and ×100 (right panels, scale bar = 100 μm) magnification. Panels A–D correspond to the study groups: (**A**) Group A (CS–HA lens), showing continuous epithelium with orderly anterior stroma and no inflammatory cells; (**B**) Group B (CS lens), showing intact epithelium with partial stromal disorganization; (**C**) Group C (Commercial BCL), showing irregular epithelial boundary and moderate inflammation; (**D**) Group D (Drops only), showing the least restoration with marked stromal disorganization and prominent inflammation.

H&E sections obtained from the central corneal wound bed at day-10 are presented in Fig. 5. In Group A, the epithelium appeared regular and continuous with a smooth surface contour and a near-physiological stratification pattern (approximately 4–5 cell layers), including an identifiable basal layer and overlying wing/superficial layers; the anterior stromal lamellae were relatively well aligned and no inflammatory cell infiltration was evident. Group B showed mild epithelial hyperplasia with increased stratification (approximately 5–6 cell layers) and less uniform anterior stromal lamellar organization with occasional scattered inflammatory cells. Group C demonstrated variable epithelial thickness with mild surface undulation (approximately 5–6 layers) and focal anterior stromal disorganization accompanied by moderate inflammatory infiltration. Group D showed the most irregular epithelial architecture, with marked epithelial thickening/hyperplasia and surface undulation (approximately 7–8 layers), together with prominent stromal disorganization and increased anterior stromal cellularity consistent with inflammation.

### Clinical observations

Transient conjunctival hyperemia occurred in 2/15 eyes (13%) in CS–HA and 3/15 (20%) in CS‑only during the first 24 h; no other lens‑related signs were observed. One bacterial keratitis occurred in the drops‑only group (day-4) and resolved with intensified antibiotics. Because this eye required protocol-deviating intensified antimicrobial therapy, it was excluded from the time-to-heal (Kaplan–Meier/log-rank) analysis (Group D: *n* = 14). By day-10, no eye exhibited corneal opacity or neovascularization. Lens displacement in Groups A–B was infrequent and did not interrupt treatment.

## Discussion

Our study demonstrates that a daily replaceable dual-drug CS–HA bandage lens significantly accelerated corneal epithelial recovery compared with standard care and the daily replaceable CS-only dual-drug lens in this rabbit epithelial debridement model. These findings support the potential utility of this platform in settings where rapid epithelial closure is clinically desirable, and warrant further studies including drug-loading/release characterization and clinical translation.

Building on our previous studies of thermosensitive, in situ-gelling CS–HA formulations for corneal chemical burns^25,26^, the present work evaluates a distinct ocular delivery format: a pre-formed CS–HA bandage contact lens. In contrast to the earlier β-glycerophosphate-based thermogels, which were administered as liquids and underwent temperature-triggered gelation on the ocular surface, the current platform is fabricated ex vivo as a lens-shaped hydrogel and therefore provides immediate corneal coverage upon placement.

These differences in physical format may influence the clinical scenarios in which each platform is most suitable. In situ gelling systems may be advantageous for irregular or extensive ocular-surface injuries because they can conform to nonuniform defects, whereas a pre-formed bandage lens may be particularly attractive for standardized epithelial defects and postoperative settings requiring immediate surface protection and structural stability. In the present rabbit mechanical debridement model, this pre-cast CS–HA lens was associated with favorable optical transparency, adequate oxygen permeability for short-term wear, and accelerated re-epithelialization, supporting its potential as a therapeutic bandage platform. Nevertheless, direct comparative studies would be required to determine whether either platform is superior for specific injury patterns or clinical indications such as PRK.

Consistent with this design rationale, complete re-epithelialization occurred within 3–4 days in the CS–HA group, compared with about 5 days for the chitosan-only lens, 6 days for the commercial bandage lens plus drops, and 8 days for drops alone. This reduction in healing time represents a clinically meaningful gain, as it could lessen postoperative discomfort, lower the risk of infection, and shorten visual recovery^27,28^. Notably, the commercial silicone-hydrogel bandage lens used with topical drops (Group C) also shortened healing time compared with drops alone (Group D) (median 6 vs. 8 days; Bonferroni-adjusted log-rank *p* = 0.001471), consistent with evidence that bandage soft lenses facilitate corneal resurfacing and reduce pain in persistent epithelial defects^27,29^.

Our results are consistent with recent advances in drug-loaded and bioactive lenses. Gelatin-based lenses encapsulating rutin have been shown to accelerate corneal wound closure^30^, and collagen-based lenses releasing acidic fibroblast growth factor improved epithelialization and reduced haze in alkali burns^31^. Extending these findings, we demonstrate that a dual-drug chitosan–hyaluronic acid hydrogel lens combining antibiotic and anti-inflammatory therapy can further enhance corneal regeneration compared with standard bandage lenses or single-polymer systems.

Several factors may account for the superior performance of the CS–HA lens. Chitosan’s positively charged, mucoadhesive backbone enhances the residence time of the polymer matrix and its co-loaded therapeutics on the ocular surface, while simultaneously providing broad-spectrum antimicrobial activity through bacterial membrane disruption and metal ion chelation^10,32–36^. Beyond its antimicrobial role, chitosan also modulates the wound microenvironment by scavenging reactive oxygen species and downregulating pro-inflammatory cytokines, thereby fostering a more regenerative milieu at the site of injury^37^. Hyaluronic acid complements these effects by enhancing tear-film stability and stimulating epithelial migration and proliferation^11,38,39^, consistent with clinical evidence that HA-based therapies accelerate epithelial closure and reduce corneal inflammation^40^. Together, chitosan and hyaluronic acid likely provided synergistic mucoadhesion and extended precorneal residence time, thereby improving surface hydration and supporting epithelial migration. In parallel, local drug availability from the dual-loaded lens likely reduced bacterial colonization and attenuated early inflammatory cascades, contributing to the non-inflamed stromal profile and orderly tissue architecture observed in the CS–HA group^41,42^. The superior outcome in the CS–HA group, compared with the standard bandage lenses or the CS-only formulation, highlights the efficacy of this specific composite platform. While we cannot exclusively isolate the independent biological role of HA from the physical changes induced by the composite blending, the final CS-HA formulation optimally supported rapid epithelial regeneration. Crucially, in vitro bioassays confirmed the functional activity of the loaded therapeutics after soaking-based loading and elution, a preservation likely supported by the passive, room-temperature loading process. Levofloxacin retained potent antibacterial inhibitory activity in agar diffusion testing against both Gram-positive and Gram-negative strains (31–33 mm inhibition zones), while the eluted betamethasone demonstrated robust anti-inflammatory efficacy by significantly suppressing macrophage TNF-α secretion. Consequently, the superior in vivo healing reflects a synergistic interplay: the intrinsic regenerative and mild anti-inflammatory properties of the CS-HA scaffold were directly augmented by the targeted pharmacological actions of the co-eluted agents. This synergy is histologically supported by the rapid restoration of a well-organized, non-inflamed stroma in the dual-drug group. Clinically, no infectious keratitis was observed in Group A during the follow-up period of this small study.

From a materials perspective, the CS–HA lens exhibited visible‑light transmittance ≥ 90% (≥ 567 nm), EWC ~ 80%, and Dk ≈ 44 Barrer (1.47 × 10⁻¹⁴ mol m⁻¹ s⁻¹ Pa⁻¹). We prioritized characterizing these ISO 18369-4^21^ aligned parameters as they are critical for a short-term bandage lens to prevent hypoxia and allow wound monitoring. While mechanical properties such as tensile modulus were not explicitly quantified, the high equilibrium water content (~ 80%) and the calculated mass swelling ratio of ~ 3.95 suggest a highly hydrated, low-modulus matrix. The stable retention of the lens on the ocular surface and the absence of mechanical irritation throughout the study further support its adequate wettability and biocompatibility for the intended short-term application. The lens was not designed to block UV, and its polymer composition (high water, ionic) corresponds to FDA Group IV^43–45^. The absence of hypoxia-related adverse findings over 10 days supports the biocompatibility of the construct in this model.

Histopathological analysis reinforced the clinical improvements by showing that eyes treated with the CS–HA lens developed a well-organized, intact epithelium without signs of neovascularization or other pathological changes, consistent with normal physiological healing^46,47^. In contrast, eyes receiving the standard bandage lens or drops-alone exhibited areas of irregular epithelium, inflammatory cell infiltration, and incipient scarring. The accelerated re-epithelialization observed with the CS–HA lens was accompanied by a well-organized and stratified epithelium, which may help reduce the risk of secondary complications such as microbial keratitis or stromal haze^4,42^. By supporting rapid and orderly surface closure, the CS–HA lens created a microenvironment favorable for regenerative corneal healing.

While the results demonstrate accelerated healing, certain aspects of the current study design warrant consideration. First, the lack of in vivo drug-free controls restricts fully isolating the pharmacological effects from the matrix’s intrinsic benefits. Second, due to tissue sample exhaustion, specific in vivo immunohistochemical validation (e.g., Ki-67, ZO-1) was omitted; however, our in vitro bioassays confirmed drug potency, and H&E sections provided robust phenotypic evidence of epithelial stratification. Third, absolute drug loading, release kinetics, and surface mechanics were not quantified, which future GMP-compliant studies will address alongside the established ISO parameters. Finally, the rapid healing rate of the rabbit mechanical debridement model does not perfectly mimic human laser-induced injuries, warranting caution in direct clinical translation.

## Conclusions

In conclusion, the daily replaceable CS–HA lens offers a biocompatible scaffold with sufficient oxygen permeability, adequate optical clarity in the mid-to-long visible spectrum, and simultaneous delivery of antimicrobial and anti-inflammatory agents, leading to the most rapid and well-structured epithelial regeneration among the tested approaches in this rabbit debridement model.

## Data Availability

The datasets generated and/or analysed during the current study are available from the corresponding author (AS) on reasonable request.
